# Alpaca Farming and Management Practices in Poland: Online Questionnaire

**DOI:** 10.3390/ani16172705

**Published:** 2026-09-01

**Authors:** Natalia Broś, Iwona Anna Walachniewicz, Joanna Kapustka, Monika Budzyńska

**Affiliations:** 1 Student Scientific Circle of Animal Behaviour, University of Life Sciences in Lublin, 20-950 Lublin, Poland; 2 Department of Animal Ethology and Wildlife Management, University of Life Sciences in Lublin, 20-950 Lublin, Poland; monika.budzynska@up.edu.pl

**Keywords:** alpaca, survey, keeping conditions, health issues

## Abstract

Alpacas have gained increasing popularity in Poland; however, no official national standards for their husbandry have yet been established. This study aimed to evaluate alpaca husbandry practices on Polish farms using an anonymous questionnaire. A total of 44 farms were included in the analysis, all of which kept alpacas, while only 9.1% also maintained llamas. A high level of adherence to preventive healthcare practices was observed, with regular deworming implemented on 90.9% of farms and vaccination reported by all respondents. Dietary supplementation was also widely practised, with vitamin D being the most frequently administered supplement (90.9%). However, the assessment of biosecurity measures revealed important deficiencies in epidemiological risk management, as only 59.3% of owners quarantined newly introduced animals before integrating them into the herd. These findings provide the first comprehensive overview of alpaca husbandry practices in Poland and may serve as a basis for future research and the development of official husbandry standards for alpacas.

## Simple Summary

Alpacas are becoming increasingly popular in Poland, yet official national standards for their husbandry have not been established. This study surveyed alpaca owners across the country to provide the first comprehensive overview of housing conditions, feeding practices, management, and health issues in Polish alpaca farms. The results showed that most alpacas are kept under conditions that generally meet their welfare needs. However, owners also reported limited availability of veterinarians with sufficient experience in alpaca medicine. These findings can help breeders and veterinarians improve alpaca care and provide a scientific basis for the development of national husbandry guidelines and welfare standards.

## 1. Introduction

The alpaca *(Vicugna pacos)* is a domesticated South American camelid (SAC) originating from the Andean regions of present-day Peru and Chile [1]. Alpacas have historically been valued as sources of fibre used in the production of high-quality textiles, characterised by exceptional fineness and thermal insulation properties [2,3].

Archaeological and genetic evidence suggests that alpaca domestication began approximately 6000–5500 years ago [2,3]. Although alpacas have a long-established presence in South America, their introduction to Europe occurred only in the late 20th and early 21st centuries. In Poland, the first alpacas were imported at the beginning of the 21st century, marking the beginning of the species’ presence in the country. Organised breeding activities developed relatively recently, with the establishment of the Polish Alpaca Breeders’ Association in 2012 [4], followed by the formation of the Association of Alpaca and Llama Breeders in 2018 [5].

In Poland, the population of alpacas is still growing, numbering about 8000 animals in May 2026 [6]. Alpacas are kept to harvest their wool, but also they are used as recreational and therapeutic animals [7]. Formal recognition of camelids (including alpacas) as farm animals was introduced only recently; in 2021 they were granted official livestock status [8]. However, within this legal act, the only provisions specifically referring to alpacas concern the requirement to maintain herd books, while no statutory standard for their husbandry, management, or welfare were established. Consequently, despite obtaining livestock status, alpaca husbandry in Poland continues to operate without comprehensive legal regulations defining minimum standards of care and management. Subsequent national regulations introduced mandatory identification and registration requirements [9]. Therefore, while administrative oversight of alpaca populations has increased, statutory provisions governing the practical conditions of alpaca husbandry and welfare remain largely absent. The absence of comprehensive statutory husbandry standards underscores the need for systematic evaluation of current alpaca husbandry practices in Poland. Such evidence is essential for assessing compliance with species-specific welfare requirements and may provide a scientific basis for the future development of husbandry standards and legislative regulations. New World camelid standards have already been developed and are in use in other countries, for example, in Germany, where the Federal Ministry of Food and Agriculture (BMEL) financed a project aimed at developing guidelines for the housing conditions of alpacas and llamas (MuD Tierschutz-Projekt Neuweltkameliden) [10]. The analysis of alpacas’ and llamas’ keeping conditions has been conducted in Germany, Hungary, Austria, and the United Kingdom [11,12,13,14,15]. These investigations assessed, among other variables, paddock and pasture size, herd size and composition, as well as aspects of veterinary management and preventive healthcare. The parameters evaluated in these studies provided a useful reference framework for assessing alpaca husbandry practices and selecting variables relevant for evaluating housing, management, and welfare conditions. The aim of this study was to investigate alpaca farming and management practices in Poland based on the animal owners’ survey with regard to housing conditions, nutrition, care management and health issues.

## 2. Materials and Methods

### 2.1. Study Design and Data Collection

The questionnaire was developed based on similar studies conducted in Germany, the United Kingdom, Switzerland, and Hungary [11,14,15,16]. The target population was alpaca owners in Poland, and the study was conducted between January and May 2026. No monetary or non-monetary incentives were offered. The questionnaire was an open survey consisting of 51 single- or multiple-choice questions with the ability to select a single or multiple response options and free-response questions, about ten questions per page on five pages overall. Not every question was mandatory; consequently, not all respondents answered every question. It covers topics such as 1. General farm information, 2. Housing conditions, 3. Alpaca nutrition, 4. Care management, and 5. Health issues. The questionnaire was distributed to alpacas’ owners by sharing the survey in five Facebook (Meta Platforms Inc., Menlo Park, CA, USA) groups for alpaca owners, with no additional formal distribution channels used. It was created as an online survey using Google Forms (Alphabet Inc., Mountain View, CA, USA). There was a possibility to change the typed answer and go back to the previous pages. Participation was voluntary, and responses were completely anonymous; the questionnaire contained no questions that would allow for the identification of the farm. Participants provided informed consent to take part in the survey. They were informed that participation was voluntary and were told how long it would take to complete. The purpose of the data collection and the intended use of the data were also explained. Only fully completed questionnaires were analysed. The sample of the questionnaire is available in the Supplementary Materials for review of the survey questions.

### 2.2. Statistical Analysis

Data analysis was performed using Microsoft Excel Online (Microsoft 365, Microsoft Corporation, Redmond, WA, USA). Statistical tests were conducted using Statistica 13.1 software (StatSoft, Kraków, Poland). Results are presented as the mean (x) with standard deviation (SD). Statistical analyses to examine associations between given variables (between the occurrence of veterinarian visits and health issues, being a breeder and health issues, and the number of intact males and fight injuries) were performed using Pearson’s chi-square test. A value of *p* < 0.05 was considered statistically significant.

## 3. Results

### 3.1. General Farm Information

**Figure 1:**
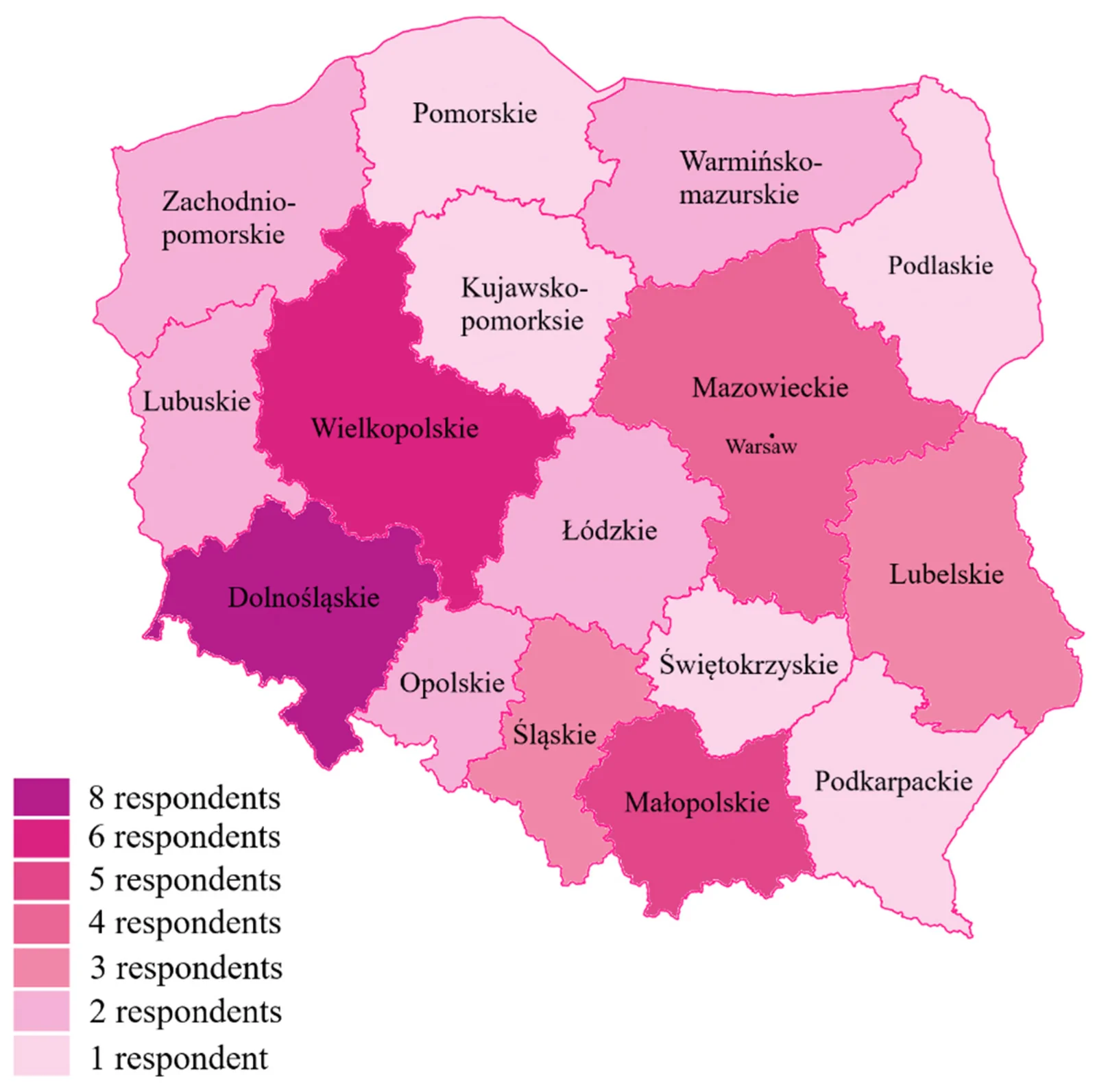
Number of alpaca farms in Polish provinces based on survey evaluation (source: author).

Forty-four respondents completed the survey. Among all the assessed farms, the largest numbers were located in the following provinces: dolnośląskie (18.2%, *n* = 8/44), wielkopolskie (13.6%, *n* = 6/44), and małopolskie (11.4%, *n* = 5/44) (Figure 1). All survey respondents kept alpacas (100%), four farms also kept llamas (9.1%, *n* = 4/44), and one farm kept camels (2.3%, *n* = 1/44). The vast majority (97.7%, *n* = 43/44) of farms kept Huacaya alpacas, with 27.2% (*n* = 12/44) also keeping Suri alpacas. Only 2.3% (*n* = 1/44) of survey respondents kept Suri alpacas exclusively.

**Figure 2:**
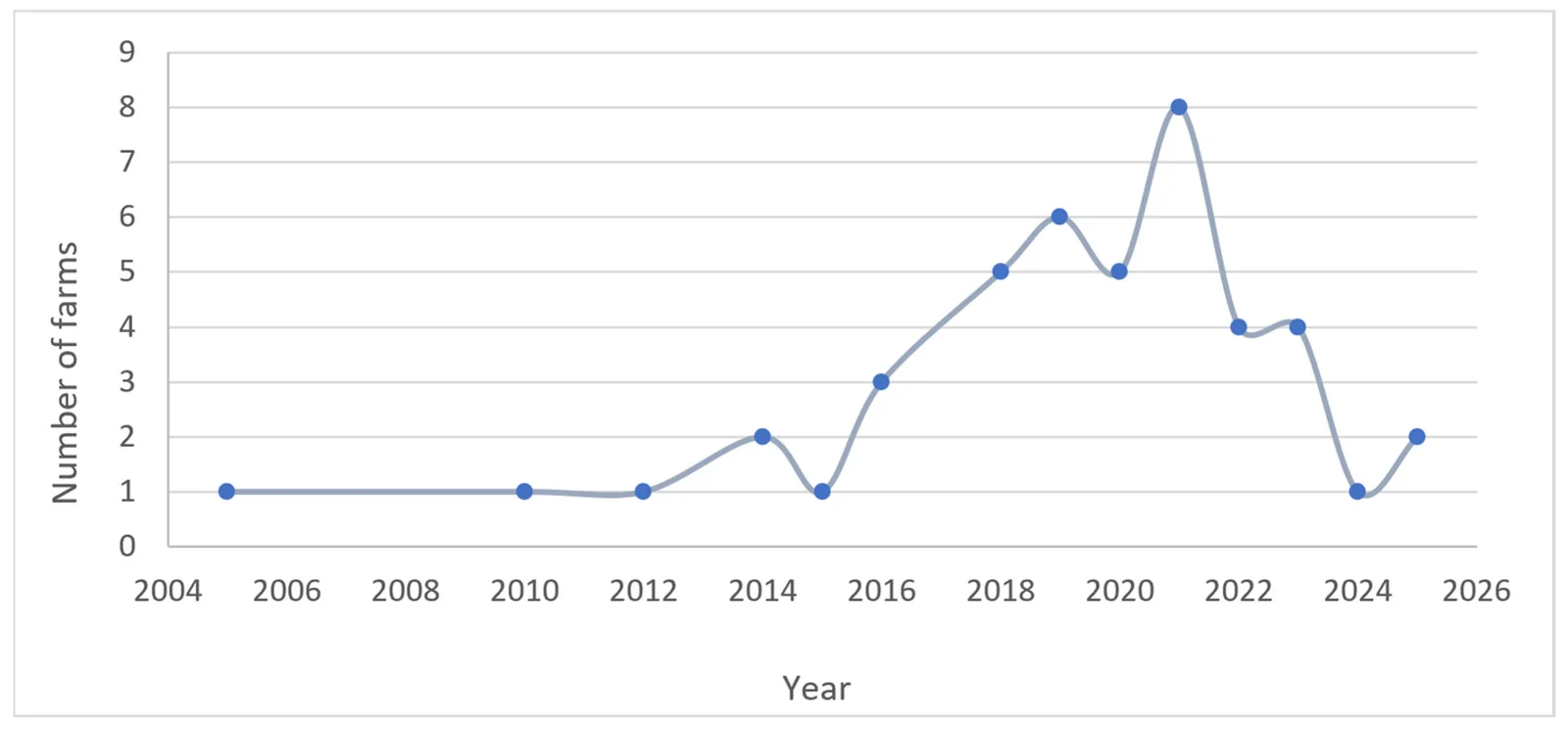
The year when owners started keeping South American Camelids.

The period when gradually more and more owners started keeping SACs was observed in the years from 2016 to 2021, and it may reflect the years of alpacas’ greatest popularity in Poland (Figure 2).

**Figure 3:**
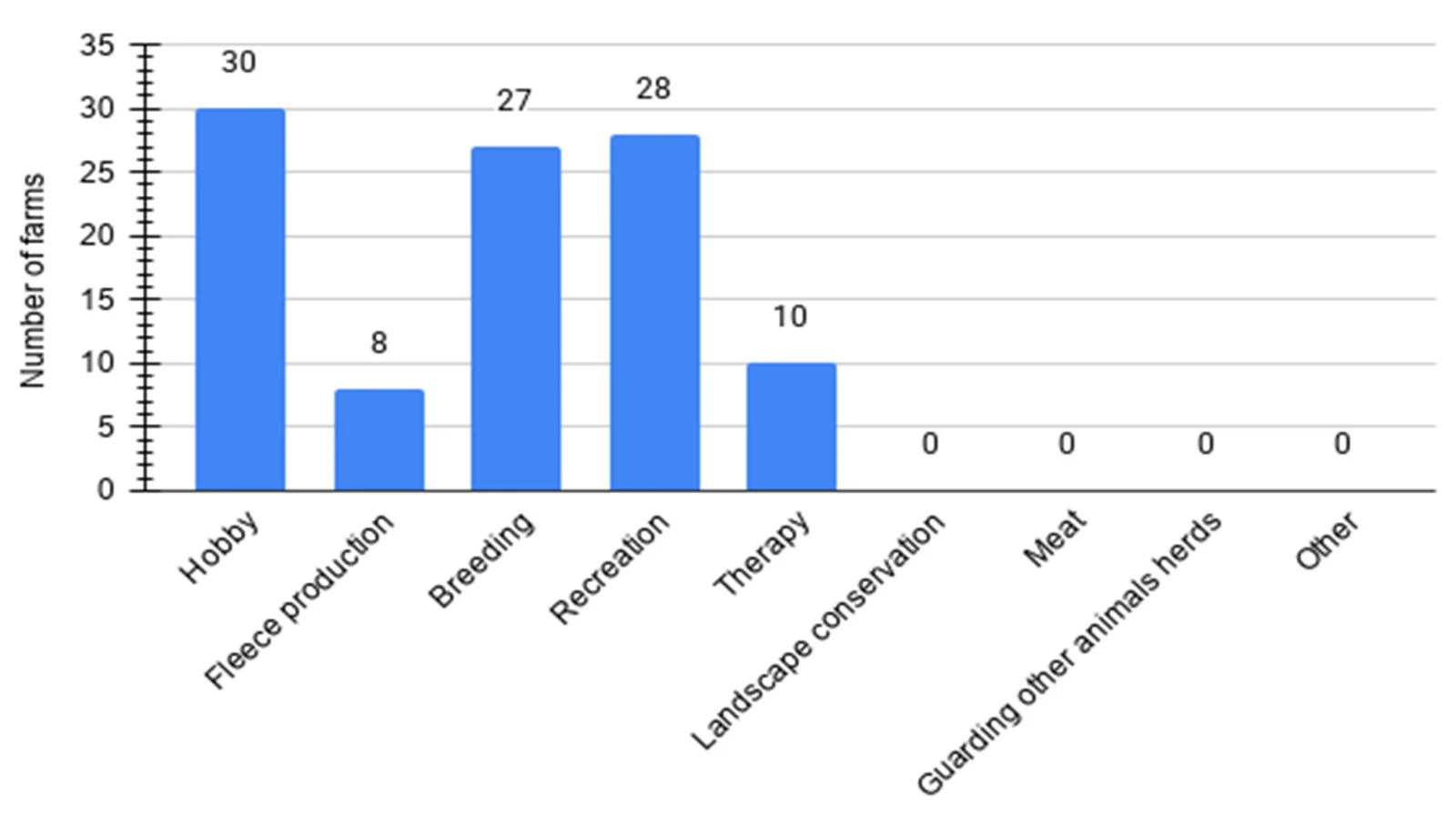
Purpose of keeping alpacas among the respondents (*n* = 44); the question allowed for the selection of multiple answers simultaneously and included an “other” option.

For 22.7% (*n* = 10/44) of respondents, keeping alpacas is their main occupation and source of income, while for 54.5% (*n* = 24/44) it is only a supplementary source of income. It is partially reflected in the purpose for keeping alpacas, with surveyed owners reporting that they keep alpacas primarily for hobby and recreation, as well as for breeding. Among the respondents, 75% (*n* = 33/44) indicated more than one purpose for keeping alpacas. The most frequently reported combinations involved hobby-based keeping, breeding, and recreation. Only 38.6% (*n* = 17/44) of farms did not keep alpacas for breeding purposes. None of the farms kept alpacas for sheep guarding, landscape conservation, or meat production (Figure 3).

### 3.2. Housing Conditions

In addition to alpacas, most farms keep poultry (43.2%, *n* = 19/44), goats (38.6%, *n* = 17/44), sheep (34.1%, *n* = 15/44), and equines (27.3, *n* = 12/44). A total of 36.4% (*n* = 16/44) of the farms do not keep any other farm animals. However, only 27.3% (*n* = 12/44) of respondents reported keeping their alpacas in direct contact with other farm animal species (goats, sheep, equines). Almost all owners (97.7%, *n* = 43/44) used more than one source of information about the keeping of SACs. Owners most often gained knowledge from other alpaca and llama breeders (88.6%, *n* = 39/44). More than three-quarters of respondents (79.5%, *n* = 35/44) searched for information online, and 75% (*n* = 33/44) attended courses.

The majority of surveyed herds consisted of females and intact males. Most animals were aged between 4 and 10 years. Regarding herd organisation, 75% of respondents (*n* = 33/44) reported keeping alpacas in multiple groups, whereas 25% (*n* = 11/44) maintained a single herd. Among farms where alpacas were divided into multiple groups, the majority of owners (86.4%, *n* = 28/33) managed groups consisting of fewer than 10 individuals, while 13.6% (*n* = 5/33) kept groups of 10–20 alpacas (Table 1). Notably, 77.8% of respondents (who kept groups of 10–20 individuals) identified themselves as breeders.

### 3.3. Alpaca Husbandry

**Figure 4:**
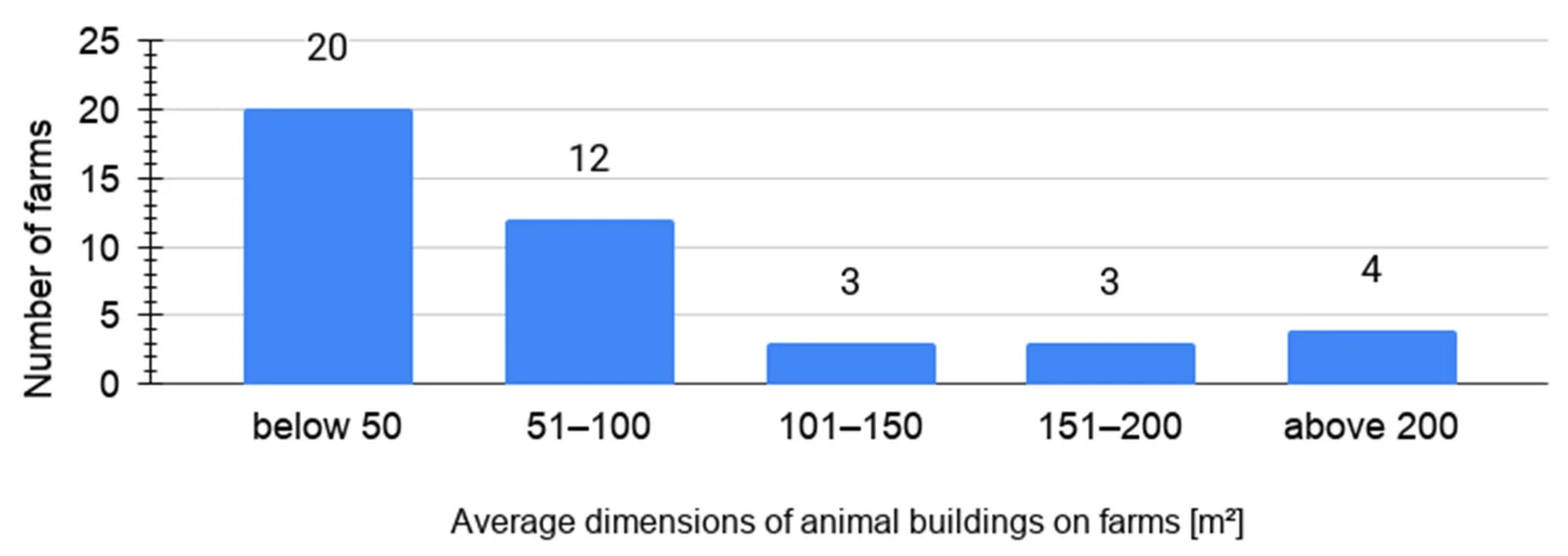
Average dimensions of animal buildings on farms (*n* = 20/44).

**Figure 5:**
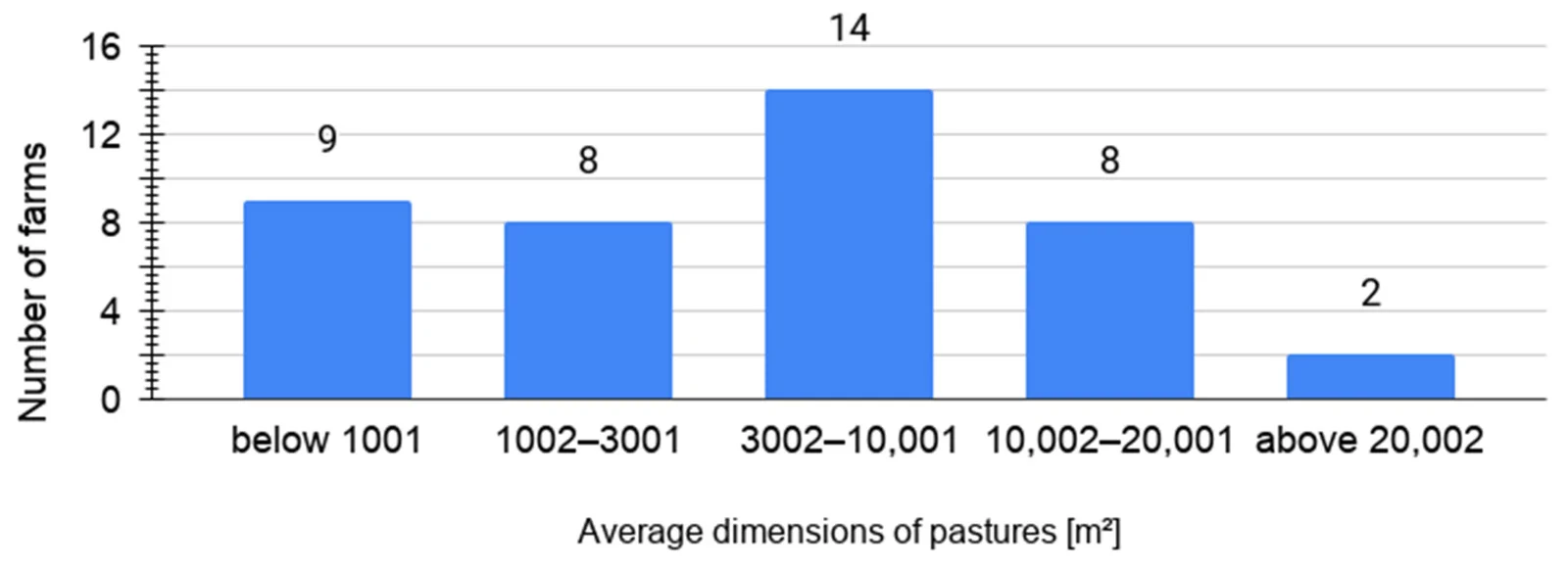
Average dimensions of pastures on farms (*n* = 42/44).

The largest group of alpaca owners (45.5%, *n* = 20/44) keeps their herds in small buildings with less than 50 m^2^ (Figure 4), but the dimensions of the pastures are medium-sized (3002 m^2^ to 10 001 m^2^) (Figure 5). Almost all owners kept their animals in a building with access to pasture (81.8%, *n* = 36/44), and some of them kept alpacas in a pasture with a shelter (9.1%, *n* = 4/44). Three survey participants kept their animals in a building with a run (6.8%, *n* = 3/44). A total of 75% (*n* = 33/44) of the owners used pasture rotation, but most did so only for certain animals. The most popular bedding type was straw (70.5%, *n* = 31/44); 25% (*n* = 11/44) of participants used hay for this purpose.

The question regarding pasture size was a free-response question and required respondents to provide a numerical value. The responses showed considerable variation, ranging from 2 m^2^ to 50,000 m^2^. To facilitate data analysis and presentation, the reported pasture sizes were subsequently categorised into predefined ranges based on their area.

### 3.4. Alpaca Nutrition

During the summer period, the diet of most herds was based on pasture grass (88.6%, *n* = 39/44) and hay (90.9%, *n* = 40/44). Other roughage was used very rarely, with the most common being unmolassed beet pulp (13.6%, *n* = 6/44) and concentrate feed (4.5%, *n* = 2/44). In winter, however, hay constituted the largest percentage of feed (100%), with unmolassed beet pulp (15.9%, *n* = 7/44) and concentrate feed (13.6%, *n* = 6/44) also being used. Most farms fed alpacas feed additives, most often declared using mineral feed (61.4%, *n* = 27/44), and vegetables and fruit (27.3%, *n* = 12/44). The majority of the farms administered vitamin D to their alpacas (90.9%, *n* = 40/44), and the animals were also frequently given vitamin E (40.9%, *n* = 18/44) and selenium (34.1%, *n* = 15/44).

### 3.5. Care Management

Most surveyed farms do not regularly introduce new animals to their herds other than their own offspring (65.9%, *n* = 29/44). About half of the surveyed owners quarantine new animals (59.3%, *n* = 16/44). A two-week quarantine period was observed most frequently (47.1%, *n* = 21/44). Most owners have origin documents for their animals (52.3%, *n* = 23/44) or only have them for some animals (25%, *n* = 11/44). A large proportion of respondents (90.9%, *n* = 40/44) tag their alpacas with transponders (microchips).

Almost all respondents indicated that they shear their alpacas every year. One respondent never shears them. Shearing on the surveyed farms was mostly performed by a professional shearer (88.6%, *n* = 39/44), mostly using the lying-down restraint method (93.2%, *n* = 41/44). A total of 15.9% (*n* = 7/44) of farms used a shearing table, and one farm sheared their alpacas standing up (2.3%, *n* = 1/44). Shortening of males’ fighting teeth was performed on 81.8% (*n* = 36/44) of farms. On farms where this procedure is performed, it is primarily performed by a veterinarian (45.5%, *n* = 20) or by the shearer (43.2%, *n* = 19/44). All respondents (100%, *n* = 44/44) reported trimming their alpacas’ nails. The largest proportion of owners (88.6%, *n* = 39/44) performed the procedure independently on their farms. Most respondents (65.9%, *n* = 29/44) reported trimming their alpacas’ nails only when necessary, whereas 22.7% (*n* = 10/44) performed the procedure more than twice per year.

All farms participating in the study vaccinated their herd (100%, *n* = 44/44) and dewormed their alpacas (90.9%, *n* = 41/44). In 52.5% (*n* = 38/44) of the farms, effectiveness checks were conducted after deworming. Detailed results regarding vaccinations, deworming and administration of additional supplements are given in Table 2.

### 3.6. Health Issues

The most common digestive health problem on surveyed farms was parasite infestation (75%, *n* = 33/44). Farms also reported diarrhoea quite frequently (36.4%, *n* = 16/44). Colic and dental problems occurred much less frequently in the surveyed herds (Table 3).

Other issues affecting the skin were most frequently reported by owners in the surveyed herds (84.1%, *n* = 37/44). Injuries from hierarchy fights occurred less frequently than skin problems, but still often. Abscesses and fungal infections were rare on farms (Table 4).

The frequency of reproductive disorders was reported to be relatively low on most farms. The most common problem occurring on the farms was difficult births (50.0%, *n* = 22/44) (Table 5). Among other diseases and symptoms observed in the surveyed farms, eye disease was the most frequently reported (18.2%, *n* = 8/44). Emaciation occurred in less than 1 case per farm (20.5%, *n* = 9/44), and neurological disorders and recumbency were observed very rarely (Table 6). As for disorders affecting the cardiovascular system, they were observed very rarely by the owners; only 13.6% (*n* = 6/44) of the surveyed owners reported pneumonia in their herd less than once a year, and 7% (*n* = 3/44) of farms observed anaemia less frequently than once a year. Cardiac disease occurred on only 4.5% (*n* = 2/44) of the surveyed farms. Symptoms and diseases affecting the musculoskeletal system were not reported on the majority of surveyed farms. Only 8 farms observed open wounds in their herds (18.2%, *n* = 8/44). Eight owners reported lameness in their animals (18.2%, *n* = 8/44); two reported limb deformities (4.5%), and one reported a broken bone (2.3%).

A statistically significant relation was found between a higher frequency of veterinary visits to the farm and the occurrence of fungal infections (*p* = 0.009, Chi^2^ = 23.584). Respondents who identified themselves as breeders reported statistically more cases of diarrhoea (*p* = 0.038, Chi^2^ = 8.421), abortions (*p* = 0.008, Chi^2^ = 9.734), difficult births (*p* = 0.001, Chi^2^ = 13.639), weak cria growth (*p* = 0.045, Chi^2^ = 6.218), as well as pneumonia (*p* = 0.020, Chi^2^ = 7.833) in their herds.

All surveyed farms were under veterinary care. The most common frequency of veterinary visits was reported to be four times a year (36.4%, *n* = 16/44). A proportion of respondents (20.5%, *n* = 9/44) stated that veterinary visits occurred on an as-needed basis. The majority of owners were not fully satisfied with the level of veterinary expertise regarding the care of SACs provided by their herd veterinarian, with only 27.3% (*n* = 12/44) describing it as satisfying or partially satisfying. In comparison, most owners self-assessed their own knowledge of major camelid diseases as good (54.5%, *n* = 24/44) or very good (20.5%, *n* = 9/44).

## 4. Discussion

Comparing the results with studies conducted in Germany, Hungary, and Austria by other researchers [11,12,14], several similarities and differences can be identified in alpaca husbandry practices across Europe. The vast majority of owners in Poland kept alpacas, much more frequently than llamas or other camelids. The observed increase in newly established farms in Poland coincides with the years of alpacas’ greatest popularity, the global COVID-19 pandemic, and the post-pandemic period. Alpacas are popular throughout the country, and the sex structure in the studied herds is quite similar to those abroad, with a clear predominance of females of breeding age. Neutered males were the least numerous, consisting mostly of older individuals, which could indicate that many owners keep alpacas for breeding purposes. Interestingly, a significant portion of Polish owners keep alpacas as their main source of income (22.7%, *n* = 10/44), while the rest earn a partial income, and only a minority report no income. These findings differ from those reported in previous European studies [11,14] and suggest that commercial activity may play a more important role among Polish alpaca owners.

The greatest similarity among the surveyed countries was observed in feeding strategies, particularly in the predominant use of forage-based diets. In Poland, alpaca nutrition is primarily based on roughage, consisting of pasture grass during the grazing season and hay during winter, whereas concentrates are used mainly as supplementary feeds. Green fodder and hay were the most frequently reported feed sources in Poland (88.6%, *n* = 39/44 and 90.9%, *n* = 40/44 of respondents, respectively), closely corresponding with findings from Germany, where 98% of respondents reported feeding green fodder and 94.1% provided hay [14]. Similarly, a study conducted in Austria confirmed that forage constitutes the basis of alpaca nutrition [12]. However, Austrian husbandry practices showed greater diversification in supplementary feeding, with a higher proportion of respondents reporting the use of concentrates (37.3%) and commercial feed products (39.8%) [12].

Differences between countries were more apparent with respect to mineral supplementation. Because Polish soils are generally deficient in several essential trace elements, including selenium, mineral supplementation is widely recommended [17]. Nevertheless, only approximately 60% (*n* = 27/44) of Polish respondents reported providing mineral supplements, compared with more than 80% of respondents in Germany. These differences may reflect variation in herd management practices, the availability of specialised feed products, or differing production objectives. The discrepancy becomes even more evident when considering specific micronutrients. Selenium supplementation largely depends on local soil conditions, whereas vitamin E requirements are influenced by forage quality and feeding management, and because Poland is recognised as a selenium-deficient region [17], supplementation with these nutrients is considered justified under local husbandry conditions [18,19]. Despite this, fewer than half of the surveyed Polish owners reported providing selenium and vitamin E supplements to their animals. In contrast, vitamin D supplementation was reported by more than 90% (*n* = 40/44) of respondents. This result is comparable to the declarations of alpaca owners from the UK (91.4%), where slightly more than half of them administer vitamin D by injection [15]. This practice is consistent with current recommendations, as alpacas are particularly susceptible to vitamin D deficiency under European conditions. Owing to their evolutionary adaptation to the high-altitude, high-UV environment of the Andes, alpacas have a limited capacity for endogenous vitamin D synthesis at the lower ultraviolet radiation levels typical of Europe, especially during the winter months [20,21,22].

Considerable differences were observed regarding the main purposes of alpaca keeping. In Poland, alpacas were primarily maintained for hobby purposes (68.2%, *n* = 30/44), followed by recreational activities (63.6%, *n* = 28/44) and breeding (61.4%, *n* = 27/44). A comparable pattern was reported in Germany, where hobby keeping was the most frequently indicated purpose (64.3%), although production-related objectives such as wool production (55.7%) and breeding (55.7%) were also highly represented [14]. In contrast, breeding was the predominant purpose in Austria (67%), followed closely by wool production (65%) and hobby-related activities (43%) [12]. Additional differences were observed in less common uses. None of the Polish respondents indicated using alpacas for landscape conservation, meat production, or the protection of other livestock species. Conversely, 19.6% of German herds were utilised for landscape conservation [14], pointing to a broader integration into multifunctional agricultural systems. Therapeutic uses also varied; while present in Poland (22.7%, *n* = 10/44) and reported in 25.9% of German herds, no respondents in Austria indicated maintaining alpacas for therapy [12,14].

Alpaca welfare standards include care procedures that should be performed regular-ly in the herd. Alpaca shearing should be done annually, and toenail checking and trimming, if necessary, is advised every 2–3 months [14]. Also, shortening the fighting teeth is recommended in 2–2.5-year-old males, as it can decrease injury risk in the herd and increase handlers’ safety during husbandry and veterinary procedures [23]. In Poland, 98% (*n* = 43/44) of alpaca owners carried out annual shearing, while 81.8% (*n* = 36/44) of farms performed shortening the males’ fighting teeth. For example, in Germany, alpacas were regularly shorn on all farms; however, only on half of the farms were the adult males’ teeth shortened [14].

Preventive health management and veterinary care protocols also highlight country differences. In Poland, vaccinations were reported by all of the respondents, and regular deworming was reported by 90.1% (*n* = 40/44) of owners. By comparison, 85.6% of German herds were vaccinated and 89.4% underwent annual deworming, whereas in Austria, deworming was highly prevalent (96.4%) but vaccination rates were much lower (39.8%). In the UK, 95.7% of respondents vaccinated the herd against clostridial disease [12,14,15]. These discrepancies may stem from variations in veterinary awareness, the availability of specialised services, and established national husbandry guidelines.

Regarding specific herd health challenges, endoparasites constituted the largest percentage of digestive system diseases in the Polish population, with an index of over 75% (*n* = 33/44). These findings are consistent with previous studies conducted in Poland that noted parasite presence in over 70% of studied individuals [24]. Moreover, earlier studies also underlined the prevalence of parasitic problems on alpaca farms in Poland [25,26]. However, unlike in Germany, where parasitic infections frequently resulted in clinical signs such as emaciation and anaemia [27,28], parasitic infections in Poland were generally asymptomatic and only rarely presented with anaemia or weight loss. Alpacas’ susceptibility to gastrointestinal parasite infection indicates the need for effective preventive programmes including regularly performed diagnostic tests, pasture rotation, maintenance of hygiene, and targeted deworming [24]. Other digestive disorders were reported infrequently in Poland, with over 50% of respondents never observing them. This observation is consistent with reports indicating that colic is uncommon in alpacas and usually represents a clinical manifestation of underlying gastrointestinal disorders [29].

Dermatological disorders appear to represent a more pressing health concern under Polish management conditions. Skin problems were reported in a high proportion of surveyed Polish herds (84.1%, *n* = 37/44), with only about 15.9% (*n* = 7/44) of owners observing no issues at all. Some farms recorded frequencies up to 10 times a year. These findings are consistent with previous reports indicating that dermatological disorders represent an important health concern in alpacas and that ectoparasitic diseases, particularly mange caused by mites, are important causes of skin lesions [30]. Conversely, the frequency of reproductive system diseases indicates overall good health in the studied population. The most frequent issues occurred during the perinatal period, particularly difficult births, which affected 22.7% (*n* = 10/44) of farms once or twice a year. Conditions such as difficult births, miscarriages, and poor cria weight gain correlated strongly with the purpose of the herd; breeders reported these conditions significantly more often than recreational owners, which is expected, given the greater reproductive activity in breeding herds.

Finally, when interpreting these differences, the historical development of the sector must be considered. The oldest alpaca breeding operation in Poland was established at the beginning of the 21st century, whereas Germany has a longer history of alpaca husbandry, with its oldest reported herd dating back to 1986 [14], and in the UK probably even earlier [31]. Alpacas gained wider popularity in Poland after 2016, with a rapid expansion and a noticeable increase in new breeding operations between 2020 and 2023. This increase coincided with the COVID-19 pandemic; however, the present study does not allow the reasons for this trend to be determined. It may have been influenced by increased interest in alternative livestock farming and rural recreational activities during isolation. In Germany, the peaks in alpaca popularity occurred earlier, in 2011 and during 2018–2019, reflecting a more gradual and sustained development of the sector compared to the recent, rapid expansion in Poland [14].

Overall, the present findings demonstrate that alpaca husbandry in Poland shares many similarities with other European countries, particularly regarding feeding practices and herd management. At the same time, differences in production objectives, preventive healthcare, and the stage of sector development highlight the need for further research and the establishment of evidence-based husbandry guidelines adapted to Polish conditions. As mentioned above, Polish legislation lacks requirements regarding the conditions for keeping SACs. Concerning the other species covered by the regulation, specifications are provided concerning, among other things, minimum housing conditions, such as pen sizes, indoor microclimatic conditions, nutritional conditions based on the needs of the individual, and the permission to perform given procedures [8] based on the Five Freedoms. Similar guidelines could be developed for SACs; however, it would be worthwhile to create a code of best practices containing recommendations regarding vaccination schedules, parasite control and prevention, and regular assessment of the herd’s health and condition. Prevention would limit the occurrence of certain problems, while regular herd control would allow for their early detection.

## 5. Limitations

Several limitations should be considered when interpreting the findings of the present study. First, the sample included 44 respondents, which was smaller than those reported in comparable studies conducted in Germany, Austria, and Hungary. Although these differences should be interpreted in relation to the varying alpaca population sizes and numbers of potential alpaca owners in each country, they may still influence the representativeness and comparability of the results. Second, participation in the survey was voluntary, and therefore the representativeness of the respondents with respect to the overall population of alpaca owners in Poland cannot be fully determined. Demographic information was not collected in this study. Participation was anonymous, and the Polish alpaca sector is relatively small, so this kind of information could increase the risk of identifying individual respondents. Our primary objective was to characterise farm management practices rather than respondent characteristics. However, the lack of demographic data restricts the ability to assess potential associations between respondent characteristics (e.g., age, education, professional background, or experience) and survey responses. Moreover, the study relied on self-reported data, and the responses may have been influenced by participants’ subjective interpretation of the questionnaire items.

## 6. Conclusions

Our study provides the first comprehensive description of alpaca farms in Poland, regarding their housing conditions, nutrition, care management and health issues. Since gaining popularity, the alpaca population in Poland has grown rapidly. However, these animals are rarely kept for their original production purposes; keeping them for hobby and recreational purposes is far more prevalent. Despite limited informational resources and a lack of specific legislation regulating camelid husbandry, owners generally succeed in providing high-quality living conditions in terms of pen size, straw bedding or access to pasture. Furthermore, they demonstrate substantial awareness of preventive healthcare, which is especially evident in consistent vaccination and deworming practices. Conversely, biosecurity measures remain underdeveloped; for instance, the low proportion of owners who quarantine new herd members indicates that this area requires significant improvement. Frequent reproductive problems, particularly difficult births, indicate that more rigorous animal selection should be implemented. Notably, the majority of respondents perceived the specialised knowledge of local veterinarians as only partially satisfactory or entirely insufficient, a deficit likely attributable to the small scale of the domestic alpaca sector and the relative novelty of the species in the country. The findings of this study can be useful for discussing current challenges in alpacas’ husbandry practices among animal owners, researchers, and veterinarians, and they can also serve as a basis for the preparation of official standards on the keeping of alpacas in Poland.

## Figures and Tables

**Table 1:** Number of respondents owning females, males, and neutered males of specific ages. The question allowed for multiple responses.

Age Group	Respondents Who Own Females	Respondents Who Own Intact Males	Respondents Who Own Neutered Males
<1 year	19 (43.2%)	18 (40.9%)	1 (2.3%)
1–3 years	27 (61.4%)	26 (59.1%)	4 (9.1%)
4–10 years	33 (75.0%)	30 (68.2%)	23 (52.3%)
>10 years	13 (29.5%)	8 (18.2%)	2 (4.5%)

**Table 2:** Care management practices on surveyed farms.

Management Practices	Type	Number of Farms
Vaccinations	*Clostridium* spp. (e.g., Covexin 10)	41 (93.2%)
	Tetanus	3 (6.8%)
	Chlamydia	1 (2.3%)
	Rabies	1 (2.3%)
	Recommended by a veterinarian	2 (2.3%)
Deworming	Once a year	1 (2.5%)
	Twice a year	5 (12.5%)
	Based on regular faecal examinations (individual samples—from each alpaca separately)	30 (75.0%)
	Based on regular faecal examinations (group samples—the entire herd)	6 (15.0%)

**Table 3:** Frequency of diseases or symptoms affecting the digestive system observed by alpaca owners.

Frequency	Gastrointestinal Parasites	Diarrhoea	Colic	Dental Problems
Never Less than once a year Once or twice a year	11 (25.0%)	28 (63.6%)	37 (84.1%)	33 (75.0%)
	22 (50.0%)	11 (25.0%)	6 (13.6%)	7 (15.9%)
	10 (22.7%)	4 (9.1%)	0 (0.0%)	2 (4.5%)
Up to 10 times a year >10 times a year	1 (2.3%)	1 (2.3%)	0 (0.0%)	1 (2.3%)
	0 (0.0%)	0 (0.0%)	0 (0.0%)	0 (0.0%)
No information	0 (0.0%)	0 (0.0%)	1 (2.3%)	1 (2.3%)

**Table 4:** Frequency of diseases or symptoms affecting the skin observed by alpaca owners.

Frequency	Abscesses	Fungal Infections	Injuries Due to Dominance Fights	Other Skin Issues
Never Less than once a year Once or twice a year	35 (79.5%)	38 (86.0%)	27 (61.4%)	7 (15.9%)
	7 (15.9%)	5 (11.4%)	11 (25.0%)	24 (54.5%)
	1 (2.3%)	1 (2.3%)	4 (9.1%)	12 (27.3%)
Up to 10 times a year >10 times a year	1 (2.3%)	0 (0.0%)	1 (2.3%)	1 (2.3%)
	0 (0.0%)	0 (0.0%)	1 (2.3%)	0 (0.0%)
No information	0 (0.0%)	0 (0.0%)	0 (0.0%)	0 (0.0%)

**Table 5:** Frequency of diseases or symptoms affecting the reproductive system observed by alpaca owners.

Frequency	Miscarriages	Stillbirths	Difficult Births	Weak Cria Growth	Congenital Disabilities	Lack of Milk	Fertility Issues
Never	30 (68.2%)	32 (72.7%)	22 (50.0%)	30 (68.2%)	35 (79.5%)	29 (65.9%)	30 (68.2%)
Less than once a year	12 (27.3%)	12 (27.3%)	12 (27.3%)	12 (27.3%)	9 (20.5%)	12 (27.3%)	11 (25.0%)
Once or twice a year	2 (4.5%)	0 (0.0%)	10 (22.7%)	2 (4.5%)	0 (0.0%)	3 (6.8%)	2 (4.5%)
Up to 10 times a year	0 (0.0%)	0 (0.0%)	0 (0.0%)	0 (0.0%)	0 (0.0%)	0 (0.0%)	0 (0.0%)
>10 times a year	0 (0.0%)	0 (0.0%)	0 (0.0%)	0 (0.0%)	0 (0.0%)	0 (0.0%)	0 (0.0%)
No information	0 (0.0%)	0 (0.0%)	0 (0.0%)	0 (0.0%)	0 (0.0%)	0 (0.0%)	1 (2.3%)

**Table 6:** Frequency of other diseases or symptoms observed by alpaca owners.

Frequency	Emaciation	Recumbency	Neurological Disorders	Eye Disorders
Never	32 (72.7%)	40 (90.9%)	37 (84.1%)	30 (68.2%)
Less than once a year	9 (20.5%)	4 (9.1%)	6 (13.6%)	8 (18.2%)
Once or twice a year	3 (6.8%)	0 (0.0%)	0 (0.0%)	4 (9.1%)
Up to 10 times a year	0 (0.0%)	0 (0.0%)	0 (0.0%)	2 (4.5%)
>10 times a year	0 (0.0%)	0 (0.0%)	0 (0.0%)	0 (0.0%)
No information	0 (0.0%)	0 (0.0%)	1 (2.3%)	0 (0.0%)

## Data Availability

The data presented in this study are on request from the corresponding author.

## Supplementary Materials

The following supporting information can be downloaded at https://www.mdpi.com/article/10.3390/ani16172705/s1, Document S1: The sample of the questionnaire.
