# Supplementary material for: Alpaca Farming and Management Practices in Poland: Online Questionnaire
Source: Animals (Basel). 2026 Sep 1;16(17):2705. doi: 10.3390/ani16172705 (PMC13564683; doi:10.3390/ani16172705)
Supplement: Supplementary file 1 [file animals-16-02705-s001.zip › Document S1. The sample of the questionnaire.pdf]

Survey on alpaca keeping conditions (English Version)

Dear Sir or Madam,

My name is **Natalia Broś**, and I am a Master's student in **Animal Science** at the **University of Life Sciences in Lublin, Poland.**

I am conducting a research study aimed at assessing the current conditions and management practices of alpaca husbandry in Poland.

The purpose of this survey is to collect statistical data on alpaca breeding and management, which will constitute research material for my Master's thesis.

All responses are anonymous and will be used exclusively for scientific and educational purposes. The questionnaire takes approximately 10–15 minutes to complete.

Your participation is greatly appreciated and will contribute valuable information to this research project.

Thank you for your time and cooperation.

Natalia Broś
Master's Student in Animal Science
University of Life Sciences in Lublin, Poland

General farm information

1. In which province is your farm located? (single choice)

- ❑ dolnośląskie
- ❑ kujawsko-pomorskie
- ❑ lubelskie
- ❑ lubuskie
- ❑ łódzkie
- ❑ małopolskie
- ❑ mazowieckie

- ❑ opolskie
- ❑ podkarpackie
- ❑ podlaskie
- ❑ pomorskie
- ❑ śląskie
- ❑ świętokrzyskie
- ❑ warmińsko-mazurskie
- ❑ wielkopolskie
- ❑ zachodniopomorskie

2. Which species of South American camelids are kept on your farm? (multiple choice)

- ❑ Alpaca
- ❑ Llama
- ❑ Other: ....

3. Which alpaca breed do you keep? (multiple choice)

- ❑ Huacaya
- ❑ Suri
- ❑ Other: ....

4. How many alpacas do you keep, and in which age and sex groups? (multiple choice)

- ❑ Females <1 year
- ❑ Females 1-3 years
- ❑ Females 4-10 years
- ❑ Females >10 years
- ❑ Males <1 year
- ❑ Males 1-3 years
- ❑ Males 4-10 years

❑ Males >10 years

❑ Neutered males <1 year

❑ Neutered males 1-3 years

❑ Neutered males 4-10 years

❑ Neutered males >10 years

5. Do you keep any other livestock? If so, which ones? (Multiple choice)

❑ I have no other farm animals

❑ Poultry

❑ Horses

❑ Sheep

❑ Goats

❑ Cattle

❑ Pigs

❑ Other: ....

6. Since when (year) have you kept South American camelids (alpacas/llamas)? (Open-ended question)

....

7. For what purpose do you keep South American camelids (alpacas/llamas)? (the term: hobby means keeping alpacas/llamas for personal/family enjoyment and companionship and the term: recreation means keeping alpacas/llamas for farm guests/visitors' activities for e.g. alpaca walks, hand feeding, photo sessions) (Multiple choice)

❑ Hobby

❑ Wool

❑ Breeding

❑ Recreation

❑ Therapy

- ❑ Landscape conservation
- ❑ Meat
- ❑ Protecting other animal herds
- ❑ Other: ....

8. Are alpacas your main source of income? (Single choice)

- ❑ Yes, they are my main income
- ❑ No, they are just additional income
- ❑ I don't earn any income from them

9. How many people are involved in the care and handling of the alpacas? (Single choice)

- ❑ 1
- ❑ 2
- ❑ 3
- ❑ 4
- ❑ 5
- ❑ More than 5

10. How do you obtain information on keeping camelids? (Multiple choice)

- ❑ Books
- ❑ Training/Courses
- ❑ Other llama/alpaca breeders
- ❑ Internet
- ❑ Veterinarian
- ❑ Online forum
- ❑ Magazines
- ❑ Family/friends
- ❑ Other: ....

Housing conditions

11. Do you keep your alpacas in several groups? (Single choice)

❑ Yes

❑ No

12. What is the average number of animals in a single group? (if "yes" in Q. 11) (Single choice)

❑ <10
❑ 10–20
❑ >20
❑ Not applicable

13. Are your alpacas kept together with another animal species (e.g., shared stall/pasture)? (Single choice)

❑ Yes

❑ No

14. With which animal species are alpacas kept? (if "yes" in Q. 13) (Multiple choice)

❑ Poultry

❑ Sheep

❑ Goats

❑ Cattle

❑ Pigs

❑ Horses

❑ Not applicable

❑ Other: ....

15. How do you keep your herd? (Single choice)

❑ Building and pasture

❑ Pasture with shelter

❑ Building with paddock

❑ Pasture only

❑ Building only

- ❑ Other: ....

16. What is the approximate size of the building [m^2]? (Open-ended question)

....

17. What type of flooring/bedding is used in the building? (Multiple choice)

- ❑ Concrete
- ❑ Straw
- ❑ Rubber mats
- ❑ Sawdust
- ❑ Hay
- ❑ Soil
- ❑ Wooden floor
- ❑ Sand
- ❑ Not applicable
- ❑ Other: ....

18. What is the approximate area of the pasture/paddock [m^2]? (Open-ended question)

....

19. How many pastures/paddocks do your alpacas have? (Single choice)

- ❑ 1
- ❑ 2
- ❑ 3
- ❑ 4
- ❑ More than 5
- ❑ Not applicable

20. Do you rotate your alpacas between pastures? (Single choice)

- ❑ Yes

- ❑ No
- ❑ Not applicable

Alpaca nutrition

21. What is the primary roughage in summer? (Multiple choice)
    - ❑ Pasture Grass
    - ❑ Hay
    - ❑ Straw
    - ❑ Haylage
    - ❑ Silage
    - ❑ Other: ....
22. What is the primary roughage in winter? (Multiple choice)
    - ❑ Hay
    - ❑ Pasture Grass
    - ❑ Straw
    - ❑ Haylage
    - ❑ Silage
    - ❑ Other: ....
23. How much of the primary roughage do you feed the animals? (Single choice)
    - ❑ As much as they want (ad libitum)
    - ❑ Limited quantity
    - ❑ Other: ....
24. Do you add feed additives to the main diet? (Multiple choice)
    - ❑ No additives
    - ❑ Mineral feed
    - ❑ Concentrate

- ❑ Vegetables
- ❑ Fruits
- ❑ Other: ....

Care management

25. Do you regularly introduce new animals (other than your own offspring)? (Single choice)

- ❑ Yes
- ❑ No

26. Where do the new animals come from? (Multiple choice)

- ❑ From various farms in Poland
- ❑ From farms in the EU
- ❑ From farms outside the EU
- ❑ From one farm in Poland
- ❑ Not applicable

27. Are new animals quarantined? (Single choice)

- ❑ No
- ❑ Yes
- ❑ Not applicable

28. How long does the quarantine last? (Single choice)

- ❑ <1 week
- ❑ 1 week
- ❑ 2 weeks
- ❑ 3 weeks
- ❑ 4 weeks
- ❑ 5 weeks
- ❑ 6 weeks

- ❑ >6 weeks
- ❑ Not applicable

29. Do you have documents regarding their origin (pedigree)? (Single choice)

- ❑ Yes
- ❑ Only for certain animals
- ❑ No

30. How are your alpacas identified? (Single choice)

- ❑ Transponder (chip)
- ❑ Ear tag
- ❑ I don't use identification
- ❑ Other: ....

31. How often do you shear the alpacas? (Single choice)

- ❑ Never
- ❑ Every other year
- ❑ Once a year
- ❑ Twice a year
- ❑ More than twice a year
- ❑ Other: ....

32. Who shears the animals? (Multiple choice)

- ❑ Professional shearer
- ❑ By my own
- ❑ Other: ....

33. How are the animals restrained during shearing? (Multiple choice)

- ❑ On the table
- ❑ Lying down
- ❑ Standing

- ❑ Other: ....

34. Do you trim the animals' nails? (Single choice)

- ❑ Yes
- ❑ No

35. Who performs the nail trimming? (Multiple choice)

- ❑ By my own
- ❑ Veterinarian
- ❑ Alpaca Breeder
- ❑ Shearer
- ❑ Other: ....

36. How often do you trim the nails? (Single choice)

- ❑ As needed
- ❑ More than twice a year
- ❑ Twice a year
- ❑ Once a year
- ❑ Every other year
- ❑ Other: ....

37. Do you shorten the fighting teeth of males (canines and the third pair of upper incisors)? (Single choice)

- ❑ Yes
- ❑ No

38. Who performs this procedure? (Multiple choice)

- ❑ Veterinarian
- ❑ By my own
- ❑ Shearer
- ❑ Breeder (from another farm)

- ❑ Not applicable
- ❑ Other: ....

39. Do you vaccinate your alpacas? (Single choice)

- ❑ Yes
- ❑ No

40. What are they vaccinated against? (Multiple choice)

- ❑ *Clostridium spp.* (e.g., Covexin 10)
- ❑ Tetanus
- ❑ Chlamydia
- ❑ Rabies
- ❑ I don't know
- ❑ Not applicable
- ❑ Other: ....

41. How often are they vaccinated? (Single choice)

- ❑ 3 times a year
- ❑ 2 times a year
- ❑ Once a year
- ❑ Every other year
- ❑ Once in a lifetime
- ❑ Not applicable
- ❑ Other: ....

42. Do you deworm your alpacas? (Single choice)

- ❑ Yes
- ❑ No

43. How are they dewormed? (Multiple choice)

- ❑ Oral administration

- ❑ Injectable administration
- ❑ Not applicable
- ❑ Other: ....

44. How often are they dewormed? (Single choice)

- ❑ Less than once a year
- ❑ Once a year
- ❑ Twice a year
- ❑ Three times a year
- ❑ Four times a year
- ❑ Based on regular fecal examinations (individual samples – from each alpaca separately)
- ❑ Based on regular fecal examinations (group samples - the entire herd)
- ❑ Not applicable

45. Do you monitor the effectiveness of deworming? (Single choice)

- ❑ No monitoring
- ❑ Yes, fecal examination after deworming
- ❑ Yes, assessment of the animal's condition
- ❑ Yes, monitoring weight gain
- ❑ Not applicable
- ❑ Other: ....

46. Which products are administered? (Multiple choice)

- ❑ Vitamin E
- ❑ Vitamin D
- ❑ Selenium
- ❑ I don't administer any
- ❑ Other: ....

Health issues

47. Indicate whether the listed conditions occur in your herd and, if so, how frequently. (Single choice)

	Never	Less than once a year	Once or twice a year	Up to ten times a year	>10 times a year	I don't know
Gastrointestinal Parasites	○	○	○	○	○	○
Diarrhoea	○	○	○	○	○	○
Colic	○	○	○	○	○	○
Dental Problems	○	○	○	○	○	○
Other issues with the digestive system	○	○	○	○	○	○
Abscesses	○	○	○	○	○	○
Fungal Infections	○	○	○	○	○	○
Injuries due to dominance fights	○	○	○	○	○	○
Other skin issues	○	○	○	○	○	○
Miscarriages	○	○	○	○	○	○
Stillbirths	○	○	○	○	○	○

Difficult Births	○	○	○	○	○	○
Weak cria growth	○	○	○	○	○	○
Congenital disabilities	○	○	○	○	○	○
Lack of Milk	○	○	○	○	○	○
Fertility issues	○	○	○	○	○	○
Emaciation	○	○	○	○	○	○
Recumbency	○	○	○	○	○	○
Neurological Disorders	○	○	○	○	○	○
Eye Disorders	○	○	○	○	○	○
Pneumonia	○	○	○	○	○	○
Heart disease	○	○	○	○	○	○
Anaemia	○	○	○	○	○	○
Fractures	○	○	○	○	○	○
Lameness	○	○	○	○	○	○
Open Wounds	○	○	○	○	○	○
Limb Deformities	○	○	○	○	○	○

48. Are there any other diseases you consider important? (Open-ended question)

....

49. How often does the veterinarian visit the farm? (Single choice)

- ❑ Once a year
- ❑ Twice a year
- ❑ Three times a year
- ❑ Four times a year
- ❑ Monthly
- ❑ Several times a month
- ❑ Other: ....

50. Rate the statement: "Veterinarians generally have sufficient knowledge to treat South American camelids." (Single choice)

- ❑ Definitely yes
- ❑ Probably yes
- ❑ Partially
- ❑ Probably not
- ❑ Definitely not
- ❑ I have no opinion

51. How do you rate your knowledge regarding the main diseases affecting camelids? (Single choice)

- ❑ Very good
- ❑ Good
- ❑ Average
- ❑ Poor
